# The association of smoking and opium consumption with the risk and prognosis of colorectal cancer: A multicenter retrospective cohort study

**DOI:** 10.1097/MD.0000000000048199

**Published:** 2026-04-24

**Authors:** Mohammad Rezazadeh, Morteza Mansourian, Ahmadreza Kheradpishe, Mahya Safiei, Abolfazl Akbari

**Affiliations:** a Student Research Committee, School of Medicine, Iran University of Medical Sciences, Tehran, Iran; b Health Promotion Research Center, Iran University of Medical Sciences, Tehran, Iran; c Colorectal Research Center, Iran University of Medical Sciences, Tehran, Iran.

**Keywords:** colorectal cancer, opium, smoking

## Abstract

Colorectal cancer (CRC) is among the leading global causes of cancer-related morbidity and mortality. While lifestyle factors such as smoking and opium use have been implicated in CRC risk and prognosis, their clinicopathological and survival associations remain unclear. This study aimed to evaluate the impact of these exposures on CRC development, tumor characteristics, and overall survival (OS). A retrospective cohort study with a total of 270 participants aged ≥45 years from 2019 to 2020 was conducted in a multicenter design. Participants were divided into 2 groups of patients with CRC who underwent elective surgery and patients who were admitted for noncancerous conditions. Exposures to smoking and opium, clinicopathological profiles, laboratory data, and survival outcomes over a 5-year follow-up were extracted retrospectively via medical records. Adjusted and unadjusted odds ratios (OR) were computed to identify predictors of CRC development using binary logistic regression analysis. Kaplan–Meier and multivariable Cox regression analyses assessed survival differences and hazard ratios by clinicopathological characteristics. Smoking was more prevalent among CRC patients than controls (*P* = .041) and was associated with higher CRC risk in univariate logistic regression (OR = 2.19, 95% confidence interval 1.01–4.72; *P* = .045), though this association was not significant after adjustment (OR = 2.26, 95% confidence interval 0.96–5.27; *P* = .059). Opium consumption showed no significant association with CRC risk (*P* = .374), except for a lower platelet count among users (*P* = .023). In multivariate analysis using Cox regression, neither smoking nor opium use was significantly associated with OS. Kaplan–Meier curves confirmed no survival differences between exposed and unexposed groups. Smoking showed a potential role in CRC development, while opium consumption was not linked to prognosis. These findings highlight lifestyle exposures as possible contributors to CRC risk, warranting further investigation in larger cohorts.

## 1. Introduction

Colorectal cancer (CRC) is the third most common form of cancer globally. According to GLOBOCAN 2022, CRC accounts for 9.6% of all new cancer cases, with an incidence of 1926,118 worldwide. It also ranks second in mortality rate with 903,859 attributed deaths, accounting for 9.3% of all cancer deaths.^[1]^ The estimated number of new cases of CRC in 2022 varied by region. With 966,399 new cases, Asia reported the highest incidence of cases of CRC. The worldwide burden of CRC is rising quickly as a result of demographic shifts, population growth, and the westernization of lifestyle choices.^[2]^ Although CRC is more common in developed countries, its incidence is rising in developing countries.^[3,4]^ A total of 43,580 new CRC cases (55.96% of which were male) were reported between 2014 and 2017 in Iran.^[5]^

In recent years, concerns have been raised that the clinicopathological profile of CRC could influence biological behaviors of the tumor. Tumor location, grade, stage, and histopathology affect the prognosis of CRC.^[6]^ Compared to other stages, early-stage colon cancer had better survival rates for disease recurrence, mortality without recurrence, and mortality after recurrence.^[7]^ The mortality rate for CRCs requiring emergency surgery is rapidly increasing because these cases typically exhibit a more advanced stage than elective cases.^[8]^ Additionally, primary CRC tumors detected by screening had a 16.6% higher 5-year overall survival (OS) after metastases. These findings highlight how early tumor and clinicopathological profile detection may lead to more accurate treatment and a better overall prognosis for CRC.^[9]^

The correlation between increased CRC prevalence and westernization underscores the significance of lifestyle in disease etiology. Previous studies have verified the association between some risk factors, such as obesity, inactivity, low socioeconomic status, smoking, and some nutritional habits, such as carbohydrates, fats, red meat, and low fiber use, with CRC risk and prognosis.^[10]^ Another factor that has been questioned is the use of opium and its derivatives. Nonetheless, it has been demonstrated to be a risk factor for a number of cancers, including those of the stomach, lungs, larynx, bladder, and esophagus.^[11]^ According to previous research, opium consumption was significantly correlated with a higher risk of CRC-related death.^[12]^ Furthermore, the effects of opium and smoking on the clinicopathological features and survival of patients with CRC have also been the subject of conflicting findings in a number of studies.^[13,14]^ According to studies, more investigation is necessary to fully understand how lowering risk factors affects the incidence of CRC.

This study was designed to explore the association of smoking and consumption of opium on the clinicopathological characteristics, development chance, and survival of CRC.

## 2. Methods and materials

**Figure 1.**
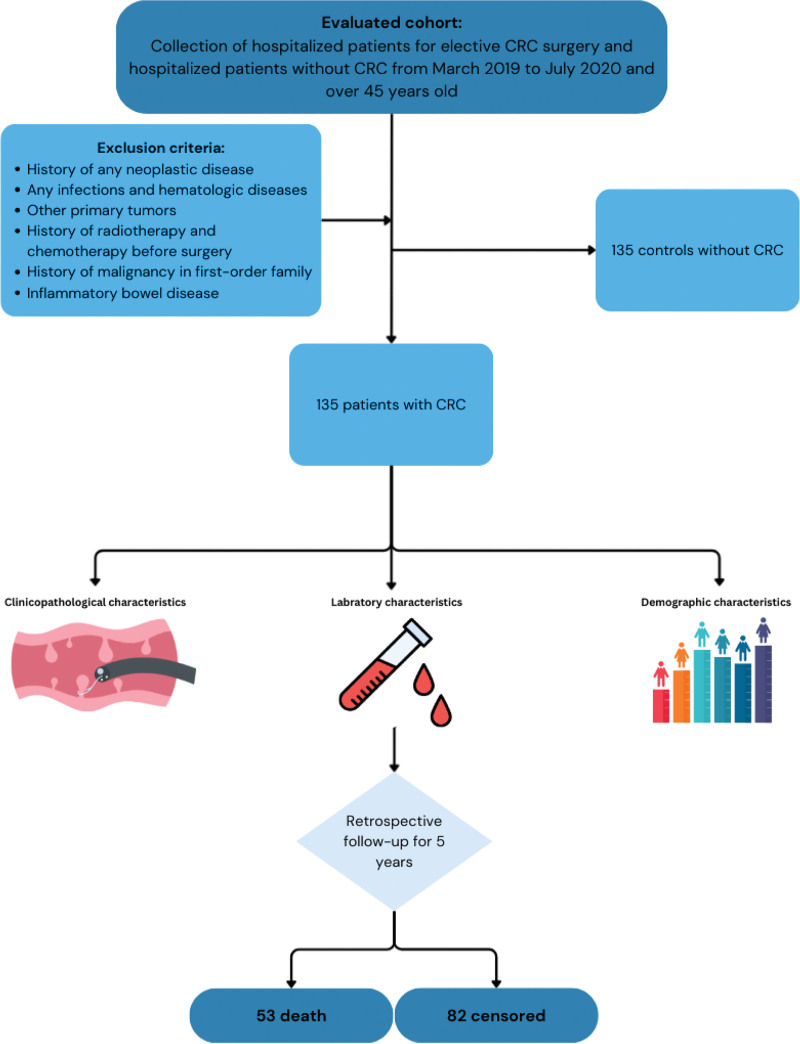
Flow chart of the sampling and data collection procedure.

This retrospective cohort study was approved with the ethics code IR.IUMS.FMD.REC.1404.112. The study followed the Strengthening the Reporting of Observational Studies in Epidemiology (STROBE) reporting guideline for cohort studies. The flowchart of the sampling and data collection procedure is shown in Figure 1.

### 2.1. Sample size calculation

The sample size in this study was computed using Power and Sample software version 3.1.2 by William D. Dupont and Walton D. Plummer for independent cohorts with 1 patient without CRC per patient with CRC. The study from Khosravizadegan Z et al was adopted as it has the most similar criteria and location to this study.^[11]^ With 80% study power, a 2-sided α of 0.05, and expected proportions (prevalence of opium consumption) in patients with CRC and patients without CRC of 0.20 and 0.05, at least 150 patients with and without CRC were required for the investigation. Based on the specified time period, the number of 135 patients with CRC and 135 patients without CRC was determined as the selected sample size.

### 2.2. Study participants

In this multicenter retrospective study, participants were all over 45 years old. Patients admitted for elective CRC surgery and hospitalized patients without CRC at 2 independent hospitals from March 2019 to July 2020 were selected.

The inclusion criteria of CRC participants were as follows: 1. All primary tumors, including codes C18–C20 based on the International Classification of Diseases, 10th Revision 2. All the chosen patients underwent elective CRC surgery.

The exclusion criteria were as follows: patients with a history of any neoplastic disease; patients with any infectious or hematologic diseases; patients with a combination of other primary tumors; patients who did undergo radiotherapy and chemotherapy before surgery; patients with a history of malignancy in first-order family; patients with inflammatory bowel disease.

### 2.3. Sampling and data collection procedure

All information in this study was collected retrospectively from medical records that were collected by self-report using in-person interviews by the trained physicians. Parameters that were recorded at baseline from participants included demographic profile, past medical history, including type 2 diabetes (T2D), hypertension (HTN), and ischemic heart disease (IHD), and smoking and opium consumption history prior to diagnosis. Aspirin at a dose of 80 mg was also collected as a drug history.

CRC stage (according to pTNM AJCC 8th edition), tumor location (proximal, distal, rectosigmoid, and overall, which means being in more than 2 of the 3 locations mentioned), grade of the tumor (well differentiated, moderately differentiated, and poorly differentiated), distant metastasis, and precursor pathology (tubular, villous, tubulovillous, and serrated) were selected as the clinicopathological profile of the tumor via colonoscopy findings and biopsy reports, according to College of American Pathologists protocol. Blood samples of patients with CRC were collected at the same time as the biopsy, including white blood cell, lymphocyte, neutrophil, platelet count, C-reactive protein, carcinoembryonic antigen, and albumin level.

### 2.4. Assessment of smoking and opium consumption status

In this study, smoking includes any form of tobacco use, whether through cigars, cigarillos, waterpipe tobacco, pipe tobacco, or smokeless tobacco products. According to similar studies, ever smoking was defined as regular smoking, daily or on most days of the week for a period of ≥6 consecutive months, for at least 12 months before CRC diagnosis. Opium consumption was defined as the injection or smoking consumption of opium and its derivatives, including raw opium (teriak), sap (shireh), burned opium (sukhteh), and heroin. Ever opium consumption was defined as consuming at least once a week for a period of ≥6 consecutive months for at least 12 months before CRC diagnosis. Both smoking and opium consumption status was categorized as never and ever smoking.

### 2.5. Follow-up

Patients with CRC were followed for 5 years. Follow-up time started at diagnosis, and the OS was defined as the interval between diagnosis and time of death. Vital status was determined from state death records, and cause of death was verified by medical records. All patients alive at the most recent follow-up were censored on that date in the survival analysis.

### 2.6. Statistical analysis

All statistical analyses were performed with SPSS software version 27.0.1 (Chicago). Descriptive statistics were used to analyze the continuous variables using mean and standard deviation. Qualitative variables were expressed as numbers and percentages. The Shapiro–Wilk test was used to check the normality of quantitative variables. To evaluate the correlation of smoking and opium consumption with characteristics of CRC patients, the chi-square and Mann-Whitney U tests were used for categorical and qualitative variables, respectively. To identify factors affecting CRC development, univariate binary logistic regression analysis was performed, and variables with a *P*-value <.05 in univariate regression were entered into multivariate regression analysis. Data were adjusted for age, sex, aspirin consumption, T2D, HTN, IHD, smoking, and opium consumption.

The trend of mortality during the study period was plotted using the Kaplan–Meier approach, and the log-rank test was used to compare the survival differences. Univariate Cox proportional regression was constructed to estimate the mortality risk by adjusting for age, sex, aspirin consumption, T2D, HTN, IHD, smoking and opium consumption, tumor location, stage, metastasis, and grade of tumor by hazard ratio. The statistical significance was considered as a *P*-value <.05 and 95% confidence intervals (CIs).

## 3. Results

### 3.1. Baseline characteristics

**Figure 2.**
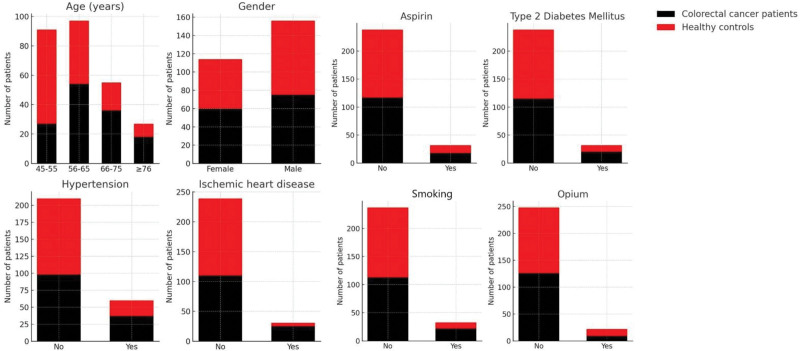
The composition of the study cohort regarding age, gender, aspirin use, type 2 diabetes, hypertension, IHD, smoking, and opium consumption by stacked bar chart. IHD = ischemic heart disease.

Table 1 shows the demographic profile of participants. Age distribution differed between patients with and without CRC (*P* <.001). Among CRC patients the most frequent age categories were 56 to 65 years (54, 40.0%) and 66 to 75 years (36, 26.7%), whereas controls were concentrated in the 45 to 55 years group (64, 47.4%). Compared with controls, CRC patients had a higher prevalence of HTN (*P* = .040) and IHD (*P* <.001). Current smoking was more frequent among patients with CRC than patients without CRC (*P* = .041). There was no significant difference in sex (*P* = .460), aspirin use (*P* = .451), T2D prevalence (*P* = .132) aspirin (*P* = .451), and opium (*P* = .374) use between patients with and without CRC. The composition of the study participants based on age, gender, use, T2D, HTN, IHD, aspirin, smoking, and opium consumption is shown in Figure 2.

**Table 1:** Characteristics of the patients with colorectal cancer and healthy controls.

Variable		Colorectal cancer patients (n = 135)	Healthy controls (n = 135)	*P*-value
Demographic characteristics				
Age (year)	45–55	27 (20.0%)	64 (47.4%)	<.001
	56–65	54 (40.0%)	43 (31.9%)	
	66–75	36 (26.7%)	19 (14.1%)	
	≥76	18 (13.3%)	9 (6.7%)	
Sex	Female	60 (44.4%)	54 (40.0%)	.460
	Male	75 (55.6%)	81 (60.0%)	
Aspirin	No	117 (86.7%)	121 (89.6%)	.451
	Yes	18 (13.3%)	14 (10.4%)	
Type 2 diabetes	No	115 (85.2%)	123 (91.1%)	.132
	Yes	20 (14.8%)	12 (8.9%)	
HTN	No	98 (72.6%)	112 (83.0%)	.040
	Yes	37 (27.4%)	23 (17.0%)	
Ischemic heart disease	No	110 (81.5%)	129 (95.6%)	<.001
	Yes	25 (18.5%)	6 (4.4%)	
Smoking	No	113 (83.7%)	124 (91.9%)	.041
	Yes	22 (16.3%)	11 (8.1%)	
Opium	No	126 (93.3%)	122 (90.4%)	.374
	yes	9 (6.7%)	13 (9.6%)	
Clinicopathological characteristics				
Tumor location	Proximal	29 (21.5%)	–	
	Distal	10 (7.4%)		
	Rectosigmoid	81 (60.0%)		
	Overall	15 (11.1%)		
Stage	1	7 (5.2%)		
	2a	22 (16.3%)		
	2b	14 (10.4%)		
	3a	26 (19.3%)		
	3b	19 (14.1%)		
	3c	19 (14.1%)		
	4	25 (18.5%)		
Metastasis	No	112 (81.4%)		
	Yes	25 (18.6%)		
Grade of tumor	Well	47 (34.8%)		
	Moderate	73 (54.1%)		
	Poor	15 (11.1%)		
Precursor pathology	Tubular	41 (30.4%)		
	Villous	32 (23.7%)		
	Tubulovillous	51 (37.8%)		
	Serrated	11 (8.1%)		
Follow outcome	Death	53 (39.3%)		
	Censored	82 (60.7%)		
Overall survival	1-yr overall survival	28 (79.3%)		
	2-yr overall survival	5 (75.6%)		
	3-yr overall survival	6 (71.1%)		
	4-yr overall survival	8 (65.2%)		
	5-yr overall survival	88 (60.7%)		
Laboratory data (Mean ± SD)				
White blood cell	9.1 ± 7.2			
Lymphocyte	1.5 ± 1.1			
Neutrophile	6.8 ± 4.1			
Platelet	220 ± 108			
C-reactive protein	46.0 ± 11.1			
Carcinoembryonic antigen	40.4 ± 20.4			
Albumin	3.5 ± 0.6			

Clinicopathological characteristics of the CRC cohort are summarized in Table 1. The most common tumor location was the rectosigmoid segment (60.0%). Stage distribution was heterogeneous, with stage 3a was the single most frequent stage (19.3%). Regarding precursor lesions, tubulovillous type was the most common (37.8%).

### 3.2. The association of smoking and opium consumption with characteristics of CRC patients

Correlation analyses between smoking, opium use and patient characteristics are reported in Table 2. Smoking was significantly associated with higher prevalence of IHD (*P* = .019). No other clinicopathological or demographic characteristics showed a statistically significant association with smoking.

**Table 2:** The correlation of smoking, alcohol, and opium consumption with characteristics of patients with colorectal cancer.

Variable	Smoking			Opium		
	Yes	No	*P*-value	Yes	No	*P*-value
Demographic characteristics						
Age (year)						
45–55	5 (22.7%)	22 (19.5%)	.062	3 (33.3%)	24 (19.0%)	.520
56–65	13 (59.1%)	41 (36.3%)		4 (44.4%)	50 (39.7%)	
66–75	1 (4.5%)	35 (31.0%)		2 (22.2%)	34 (27.0%)	
≥75	3 (13.6%)	15 (13.3%)		0 (0.0%)	18 (14.3%)	
Sex						
Male	12 (54.5%)	63 (55.8%)	.917	4 (44.4%)	71 (56.3%)	.487
Female	10 (45.5%)	50 (44.2%)		5 (55.6%)	55 (43.7%)	
Aspirin						
No	18 (81.8%)	99 (87.6%)	.465	7 (77.8%)	110 (87.3%)	.417
Yes	4 (18.2%)	14 (12.4%)		2 (22.2%)	16 (12.7%)	
Type 2 diabetes						
No	18 (81.8%)	97 (85.8%)	.627	8 (88.9%)	107 (84.9%)	.746
Yes	4 (18.2%)	16 (14.2%)		1 (11.1%)	19 (15.1%)	
HTN						
No	17 (77.3%)	81 (71.7%)	.591	7 (77.8%)	91 (72.2%)	
Yes	5 (22.7%)	32 (28.3%)		2 (22.2%)	35 (27.8%)	
Ischemic heart disease						
No	14 (63.6%)	96 (85.0%)	.019	8 (88.9%)	102 (81.0%)	.554
Yes	8 (36.4%)	17 (15.0%)		1 (11.1%)	24 (19.0%)	
Clinicopathological characteristics						
Tumor location						
Proximal	1 (4.5%)	9 (8.0%)	.620	1 (11.1%)	14 (11.1%)	.057
Distal	6 (27.3%)	23 (20.4%)		4 (44.4%)	25 (19.8%)	
Rectosigmoid	14 (63.6%)	67 (59.3%)		2 (22.2%)	79 (62.7%)	
Overall	1 (4.5%)	14 (12.4%)		1 (11.1%)	14 (11.1%)	
Stage						
1	1 (4.5%)	6 (5.3%)	.713	1 (11.1%)	6 (4.8%)	.796
2	6 (27.3%)	33 (29.2%)		3 (33.3%)	36 (28.6%)	
3	9 (40.9%)	55 (48.7%)		4 (44.4%)	60 (47.6%)	
4	6 (27.3%)	19 (16.8%)		1 (11.1%)	24 (19.0%)	
Metastasis						
No	16 (72.7%)	96 (85.0%)	.163	8 (88.9%)	104 (82.5%)	.625
Yes	6 (27.3%)	17 (15.0%)		1 (11.1%)	22 (17.5%)	
Grade of tumor						
Well	10 (45.5%)	37 (32.7%)	.519	5 (55.6%)	42 (33.3%)	.297
Moderate	10 (45.5%)	63 (55.8%)		4 (44.4%)	69 (54.8%)	
Poor	2 (9.1%)	13 (11.5%)		0 (0.0%)	15 (11.9%)	
Precursor pathology						
Tubular	8 (36.4%)	33 (29.2%)	.330	2 (22.2%)	39 (31.0%)	.443
Villous	7 (31.8%)	25 (22.1%)		4 (44.4%)	28 (22.2%)	
Tubulovillous	7 (31.8%)	44 (38.9%)		2 (22.2%)	49 (38.9%)	
Serrated	0 (0.0%)	11 (9.7%)		1 (11.1%)	10 (7.9%)	
Follow outcome						
Death	7 (31.8%)	46 (40.7%)	.435	3 (33.3%)	50 (39.7%)	.706
Censored	15 (68.2%)	67 (59.3%)		6 (66.7%)	76 (60.3%)	
Survival						
1-yr survival	3 (13.6%)	25 (22.1%)	.916	1 (11.1%)	27 (21.4%)	.623
2-yr survival	1 (4.5%)	4 (3.5%)		0 (0.0%)	5 (4.0%)	
3-yr survival	1 (4.5%)	6 (5.3%)		1 (11.1%)	6 (4.8%)	
4-yr survival	1 (4.5%)	6 (5.3%)		0 (0.0%)	7 (5.6%)	
5-yr survival	16 (72.7%)	72 (63.7%)		7 (77.8%)	81 (64.3%)	
Laboratory data (Mean ± SD)						
White blood cell	8.5 ± 4.0	9.3 ± 10.1	.495	9.2 ± 9.6	8.4 ± 3.1	.571
Lymphocyte	1.5 ± 0.8	1.6 ± 1.4	.791	1.6 ± 1.4	1.4 ± 0.7	.566
Neutrophile	6.2 ± 3.7	7 ± 8.9	.591	6.8 ± 8.5	6.4 ± 3.2	.322
Platelet	229 ± 95	218 ± 111	.261	216 ± 108	255 ± 99	.023
C-reactive protein	45.4 ± 7.4	46.1 ± 11.9	.650	46.0 ± 11.2	45.8 ± 10.2	.877
Carcinoembryonic antigen	34.6 ± 13.4	41.9 ± 67.5	.644	41.1 ± 63.61	33.7 ± 14.48	.521
Albumin	3.6 ± 0.5	3.4 ± 0.6	.132	3.5 ± 0.62	3.6 ± 0.5	.167

SD = standard deviation.

The only statistically significant laboratory association was between opium use and platelet count, which shows opium users had a lower mean platelet count compared with nonusers (*P* = .023). No significant associations were observed between opium use and other clinicopathological or demographic characteristics.

### 3.3. Predictive value of smoking and opium consumption for CRC development

**Figure 3.**
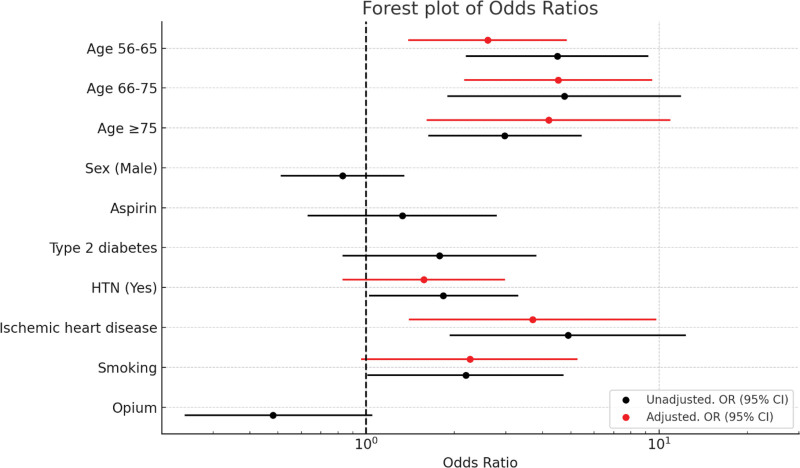
Forest plot of univariate and multivariate analysis of age, gender, aspirin use, type 2 diabetes, hypertension, IHD, smoking, and opium use as the predictors of CRC. CRC = colorectal cancer, IHD = ischemic heart disease.

Results of the binary logistic regression are shown in Table 3. In unadjusted analyses, smoking was associated with higher odds of CRC (OR = 2.19, 95% CI 1.01–4.72; *P* = .045), while opium use showed a borderline inverse association (OR = 0.43, 95% CI 0.19–1.00; *P* = .051). However, after adjustment, smoking was no longer statistically significant (OR = 2.26, 95% CI 0.96–5.27; *P* = .059). Increasing age remained a strong independent predictor of CRC in adjusted models. IHD also remained independently associated with CRC in the adjusted model (OR 3.70, 95% CI 1.40–9.79; *P* = .008). HTN was significant in unadjusted analysis but lost significance after adjustment (Fig. 3).

**Table 3:** Results of binary regression to identify colorectal cancer (patients vs healthy) predictors.

Variable		Unadjusted OR (95% CI)	*P*-value	Adjusted OR (95% CI)	*P*-value
Age (year)	45-55	Reference	–	Reference	–
	56–65	4.49 (2.19–9.18)	<.001	2.600 (1.39–4.84)	.003
	66–75	4.74 (1.89–11.87)	.001	4.528 (2.16–9.45)	<.001
	≥75	2.97 (1.63–5.43)	<.001	4.201 (1.61–10.91)	.003
Sex	Female	Reference	–	–	–
	Male	0.83 (0.51–1.35)	.460	–	–
Aspirin	No	Reference	–	–	–
	Yes	1.33 (0.63–2.79)	.452	–	–
Type 2 diabetes	No	Reference	–	–	–
	Yes	1.78 (0.83–3.81)	.136	–	–
HTN	No	Reference	–	Reference	–
	Yes	1.83 (1.02–3.30)	.042	1.57 (0.83–2.98)	.165
Ischemic heart disease	No	Reference	–	Reference	–
	Yes	4.88 (1.93–12.34)	.001	3.70 (1.40–9.79)	.008
Smoking	No	Reference	–	Reference	–
	Yes	2.19 (1.01–4.72)	.045	2.26 (0.96–5.27)	.059
Opium	No	Reference	–	–	–
	yes	0.43 (0.19–1.00)	.051	–	–

### 3.4. Prognostic value of smoking and opium consumption for CRC OS

The results of multivariate Cox regression analysis are shown in Table 4. In the present study, the 1-, 2-, 3-, 4-, and 5-year OS rates were 79.3%, 75.6%, 71.1%, 65.2%, 60.7%, respectively. According to the survival analysis, the mean (± SD) OS for all cases was 44.12 ± 23.28 months. To determine the independent prognostic factors for 5-year OS, a multivariate Cox regression analysis was performed, which showed that none of the baseline characteristics that entered the model were significantly associated with OS in patients with CRC, except for metastasis (HR = 2.20, 95% CI 1.02–4.73; *P* = .045).

**Table 4:** Results of cox regression to identify overall survival predictors.

Variable		Unadjusted HR (95% CI)	*P*-value
Age (year)	≥65	Reference	–
	<65	1.05 (0.60–1.84)	.846
Sex	Female	Reference	–
	Male	0.83 (0.48–1.44)	.514
Aspirin	Male	Reference	–
	Female	0.33 (0.10–1.08)	.069
Type 2 diabetes	No	Reference	–
	Yes	1.57 (0.78–3.14)	.198
HTN	No	Reference	–
	Yes	1.31 (0.72–2.37)	.365
Ischemic heart disease	No	Reference	–
	Yes	0.97 (0.48–1.94)	.943
Smoking	No	Reference	–
	Yes	0.52 (0.20–1.31)	.166
Opium	No	Reference	–
	yes	0.24 (0.03–1.76)	.163
Tumor location	Proximal and distal	Reference	–
	Rectosigmoid and overall	1.20 (0.64–2.26)	.558
Stage	1 and 2	Reference	–
	3 and 4	1.65 (0.88–3.11)	.116
Metastasis	No	Reference	–
	Yes	2.70 (1.47–4.95)	.001
Grade of tumor	Well and moderate	Reference	–
	Poor	0.66 (0.23–1.83)	.429

CI = confidence interval, HR = hazard ratio.

**Figure 4.**
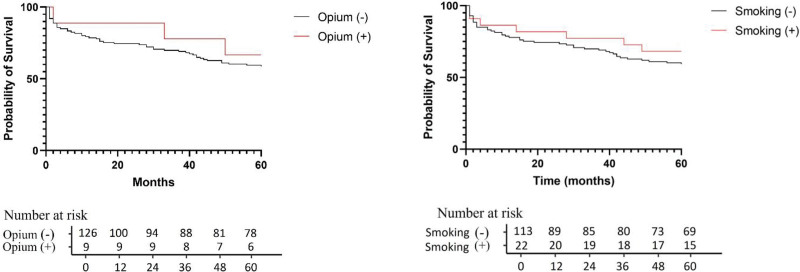
Kaplan–Meier curve of smoking and opium use in patients with CRC. CRC = colorectal cancer.

Figure 4 shows the survival differences between smokers and nonsmokers, and opium users and nonusers. According to the Kaplan–Meier survival curves, the OS of the smokers (*P*-value = 0.439) and opium users (*P*-value = 0.588) was not significantly different from nonuser patients.

## 4. Discussion

This study highlights the association of smoking and opium consumption with the clinicopathological characteristics, development chance, and survival of CRC. Opium use represents a distinctive and highly prevalent exposure in Iran, where consumption rates are among the highest globally.^[15]^ Unlike most Western countries, where opioid use is largely limited to illicit drugs or prescription misuse, in Iran opium has long-standing cultural roots and remains commonly used, particularly among older adults and in certain provinces.^[16]^ Beyond its well-established role in addiction and chronic disease, accumulating evidence links opium to an increased risk of several gastrointestinal malignancies, including cancers of the esophagus, stomach, bladder, pancreas, and colorectum.^[17]^ This high background prevalence makes Iran a uniquely important setting to investigate the relationship between opium use and CRC, as findings from such studies have the potential to inform both cancer prevention and substance use policies in the region.

Among the analyses performed, most associations were insignificant; however, several notable findings emerged. Smoking was linked to increased odds of developing CRC in univariate analysis, while age showed a strong and consistent independent effect, with risk rising steadily across categories. IHD also remained a significant predictor after adjustment, suggesting that cardiovascular comorbidities may overlap with or amplify CRC risk pathways. HTN was associated with CRC in univariate analysis but lost significance in multivariate models, and T2D showed no significant relationship. These results emphasize the importance of considering both modifiable and non-modifiable CRC risk factors. Modifiable factors include smoking, obesity, sedentary lifestyle, unhealthy diet, and psychological stress, while non-modifiable factors encompass age, gender, genetic predisposition, family history of CRC, intestinal microbiota, and personal history of other diseases.^[18]^ Together, our findings underscore that age was the strongest independent predictor in this cohort, and that comorbid conditions such as IHD may warrant closer attention in CRC risk stratification.

In our cohort, clinicopathological features such as tumor site, stage, and grade followed patterns similar to those reported in other studies. Importantly, smoking and opium use showed little influence on these characteristics. Opium use was not significantly associated with tumor stage or grade, while smoking demonstrated only a modest link with precursor pathology. These findings are consistent with prior studies reporting no strong relationship between smoking and tumor grade, although some have noted a tendency toward left-sided disease in smokers.^[6,19]^ Overall, our results suggest that lifestyle exposures such as smoking and opium may play a greater role in CRC development than in shaping the clinicopathological features.

While previous reports suggested possible associations between opioid use and clinicopathological features such as tumor grade, our analysis did not demonstrate such relationships. Opium use was not significantly associated with tumor stage, grade, or survival, though it was linked with a lower platelet count. The absence of an observed effect may be due to self-reported exposure, regional variability in consumption patterns, and small sample size. Although opium consumption has been reported as a dose-dependent risk factor for CRC in some meta-analyses,^[14]^ our study did not find a direct link. Further research is required to clarify potential mechanistic pathways, including the impact of opium on alteration of microbiome composition, intestinal epithelium integrity, modulation of the immune system, induction of apoptosis, and inhibition of angiogenesis.^[20]^

Although the association between smoking and CRC became insignificant after multivariate analysis, univariate analysis identifies smoking as a risk factor for CRC, as similar studies do.^[21]^ Smoking exposes one to a variety of over 7000 harmful chemicals, including at least 70 known carcinogens that can impact almost all of the body’s organ systems. Benzene, polycyclic aromatic hydrocarbons, nitrosamines, and heterocyclic amines are among the carcinogens found in cigarette smoke. These substances can enter the bloodstream or be directly consumed by the colorectal mucosa, and they can directly cause cancer in the colon and the rectum. Based on evidence, smoking is linked to colorectal adenomas, indicating that it likely contributes to early carcinogenesis in the colon and the rectum.^[22]^

In survival analysis, metastasis was the only factor significantly associated with reduced OS, confirming its central role as the main prognostic determinant in CRC. Smoking and opium use showed no significant effect on OS in our cohort. The few studies that have examined the association of opium on survival in patients with cancer have found shorter survivals in users, although they suggest that further studies are necessary.^[23,24]^ The 5-year relative survival rates for colon and rectum cancer based on the Surveillance, Epidemiology, and End Results database have been reported to be 63% and 67%, respectively.^[25]^ In a large study examining the association between prediagnostic smoking and CRC survival, depending on the number of pack-years smoked, both current and former smoking were linked to worse OS. Current smokers had a lower CRC-specific survival rate, but not former smokers.^[26]^ These results suggest that, while lifestyle exposures may contribute more strongly to CRC development, disease progression and survival are primarily driven by tumor biology and metastatic status.

This study has several limitations that should be acknowledged. Smoking and opium consumption were self-reported, which may introduce recall or reporting bias. The study lacked detailed information on dose, duration, and type of exposure, preventing more granular subgroup analyses. The relatively small sample size limited statistical power, particularly for assessing less common exposures such as opium use. As a 2-center study, the results may not be fully generalizable to other populations with different demographic or lifestyle profiles. Also, residual confounding cannot be excluded, as unmeasured variables such as dietary habits, physical activity, and genetic predispositions may have influenced the associations observed. Another important limitation relates to the potential for collider bias inherent in hospital-based case–control studies. Because our controls were selected from hospitalized patients admitted for noncancer conditions, they may have a higher prevalence of certain exposures such as smoking, alcohol consumption, or opium use compared to the general population.

Finally, identifying high-risk individuals or groups for CRC can help refine primary prevention strategies and enhance the effectiveness of screening programs. Risk stratification incorporating age, smoking, cardiovascular comorbidities such as IHD, and family history may improve detection of high-risk populations. All organizations emphasize the need to consider both individual and community-level factors when designing CRC prevention and control programs, and the results of this study may contribute to such efforts.^[27,28]^ Further studies with larger sample sizes and more detailed exposure assessment are warranted to clarify the complex associations between smoking, opium, comorbidities, and CRC.

## 5. Conclusion

The findings of this study suggest that the relationship between smoking, opium consumption, and CRC is more complex than previously assumed. Although smoking was more prevalent among CRC patients and appeared as a potential risk factor in univariate analysis, its effect diminished once major confounders such as age and comorbidities were considered, raising the possibility that smoking may act as a facilitator in the presence of other biological or cardiovascular risk pathways rather than an independent carcinogen in this context. Opium, despite its recognized carcinogenic role in several gastrointestinal cancers, showed no association with CRC risk or prognosis here, apart from an unexpected reduction in platelet count among users. This observation points toward possible hematologic or immunologic mechanisms that may not directly translate into tumor progression. Clinically, the results emphasize that while lifestyle factors remain critical in CRC prevention, prognostic outcomes are still overwhelmingly dictated by tumor biology, particularly metastasis, and not by smoking or opium exposure alone.

## Acknowledgments

We extend our sincere appreciation to the study hospitals staff for their invaluable support and collaboration throughout this study.

## Author contributions

**Conceptualization:** Ahmadreza Kheradpishe.

**Data curation:** Ahmadreza Kheradpishe, Mahya Safiei.

**Formal analysis:** Mohammad Rezazadeh.

**Investigation:** Mohammad Rezazadeh, Mahya Safiei.

**Methodology:** Mohammad Rezazadeh.

**Project administration:** Mohammad Rezazadeh, Abolfazl Akbari.

**Resources:** Mahya Safiei, Abolfazl Akbari.

**Supervision:** Morteza Mansourian, Abolfazl Akbari.

**Validation:** Morteza Mansourian.

**Writing – original draft:** Mohammad Rezazadeh, Ahmadreza Kheradpishe.

**Writing – review & editing:** Morteza Mansourian, Abolfazl Akbari.
